# Interferometric analysis of laser-driven cylindrically focusing shock waves in a thin liquid layer

**DOI:** 10.1038/s41598-016-0032-1

**Published:** 2016-12-23

**Authors:** David Veysset, Alexei A. Мaznev, Thomas Pezeril, Steven Kooi, Keith A. Nelson

**Affiliations:** 1 Department of Chemistry, Massachusetts Institute of Technology, Cambridge, Massachusetts 02139 USA; 2 Institute for Soldier Nanotechnologies, Massachusetts Institute of Technology, Cambridge, Massachusetts 02139 USA; 3 Institut Molécules et Matériaux du Mans, UMR CNRS 6283, Université du Maine, Le Mans, 72085 France

## Abstract

Shock waves in condensed matter are of great importance for many areas of science and technology ranging from inertially confined fusion to planetary science and medicine. In laboratory studies of shock waves, there is a need in developing diagnostic techniques capable of measuring parameters of materials under shock with high spatial resolution. Here, time-resolved interferometric imaging is used to study laser-driven focusing shock waves in a thin liquid layer in an all-optical experiment. Shock waves are generated in a 10 µm-thick layer of water by focusing intense picosecond laser pulses into a ring of 95 µm radius. Using a Mach-Zehnder interferometer and time-delayed femtosecond laser pulses, we obtain a series of images tracing the shock wave as it converges at the center of the ring before reemerging as a diverging shock, resulting in the formation of a cavitation bubble. Through quantitative analysis of the interferograms, density profiles of shocked samples are extracted. The experimental geometry used in our study opens prospects for spatially resolved spectroscopic studies of materials under shock compression.

## Introduction

Traditional shock wave studies typically involve a large scale experiment such as that performed with a gas gun^1,2^. Using short pulse lasers to generate shock waves makes it possible to conduct benchtop experiments on tiny samples and can be combined with ultrafast laser spectroscopic techniques for time-resolved spectroscopy of matter under shock loading^3–7^. A typical laser shock experiment reproduces the basic geometry of a traditional gas gun experiment: a planar shock wave produced either directly by laser ablation^8–10^ or by the impact a tiny flyer plate^11,12^ is probed optically from the opposite side. If the material is opaque, only the motion of the back surface can be measured. In a transparent material, a wide range of optical probes can be used; however, an optical probe integrates through the thickness of the material; consequently, spatial and temporal resolution can only be achieved by using thin multilayer film samples^3^. Thus the typical laser shock geometry is restricted to studying the interaction of planar shock waves with planar layered samples, which limits the possibilities for the exploration of shock-matter interactions.

We are exploring an alternative approach to laser-driven shock experiments, in which the shock wave propagates laterally within a thin layer of material confined between rigid walls^13–15^. The confined material layer is amply accessible for optical diagnostics, enabling the direct visualization of micro-shock waves. This approach is especially beneficial for studying samples or shock waves of more complex than planar geometry, such as focused shock waves^13^, which are of particular relevance to medical applications such as extracorporeal shock wave lithotripsy for stone fragmentation^16–18^. This methodology, because it enables full-field visualization of un-shocked and shocked regions, can also be implemented to study shock interaction with complex objects under complex geometries including micro-/nano-structured materials^19^ and biological cells.

The next stage in advancing the “in-plane” laser shock experiment is to develop a range of spectroscopic probes for studying material under shock. An interferometric probe for density measurements is the logical first step in this direction^20^. Interferometry has been extensively used to study shock wave propagation in 3D^21–24^; it is also particularly well suited to study 2D propagation of shock waves confined to a thin layer. The purpose of this work was to put previously demonstrated interferometric measurements^13,14^ on a quantitative basis by developing methodology for measuring density profiles of in-plane propagating shock waves^25^.

## Results

**Figure 1:**
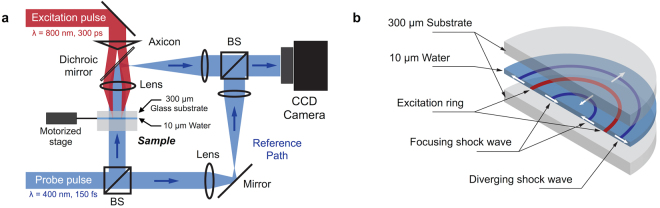
(**a**) Excitation pulse is focused into a ring in the plane of the water layer using an axicon-lens configuration. Interferometric imaging is performed in the Mach-Zehnder configuration using a variably delayed probe pulse. The probe pulse is split into two arms and recombined using two beamsplitters (BS). The sample plane is imaged onto a CCD camera using a two-lens telescope. Since a single probe pulse is used for imaging and the sample is permanently altered (with long lasting bubbles at the excitation ring) after each excitation laser shot, the sample has to be moved to an undisturbed area after every shot using a motorized stage. (**b**) Cutaway-view representation of the sample. After laser absorption by the carbon nano-particles, two counter-propagating shock waves are launched and remain confined in the plane of the sample.

Shock waves were generated by focusing a 300 ps, 800 nm excitation laser pulse delivered by an amplified Ti:sapphire system into a 10 µm-thick liquid layer. The thin liquid layer consisted of a suspension of carbon nanoparticles in water (~2 wt% carbon concentration). The layer was confined between two glass windows (300 µm thick) separated by a polymer spacer (see Methods). The excitation intensity profile was shaped into a ring in the plane of the liquid layer by using a 0.5° axicon and a 3 cm focal length achromatic doublet as described in ref. 13 and shown in Fig. 1a. The laser ring had a 190 µm diameter and a 10 µm width. As illustrated in Fig. 1b, following laser absorption by the carbon nano-particles and subsequent vaporization generating high pressure, two counter-propagating shock waves were launched and propagated laterally in the liquid layer: the inward-propagating wave that converged towards the center and the outward-diverging wave. The shock confinement in the liquid was ensured by the impedance mismatch between the liquid and the solid glass substrates. Interferometric images capturing the shock wave propagation were acquired using a Mach-Zehnder interferometer configuration and a 180-fs, 400-nm, variably-delayed probe pulse derived from the same laser system. By comparing the interferograms obtained before laser excitation and after a given delay, we were able to directly extract the change in refractive index induced by the change in the liquid density following the shock front.

**Figure 2:**
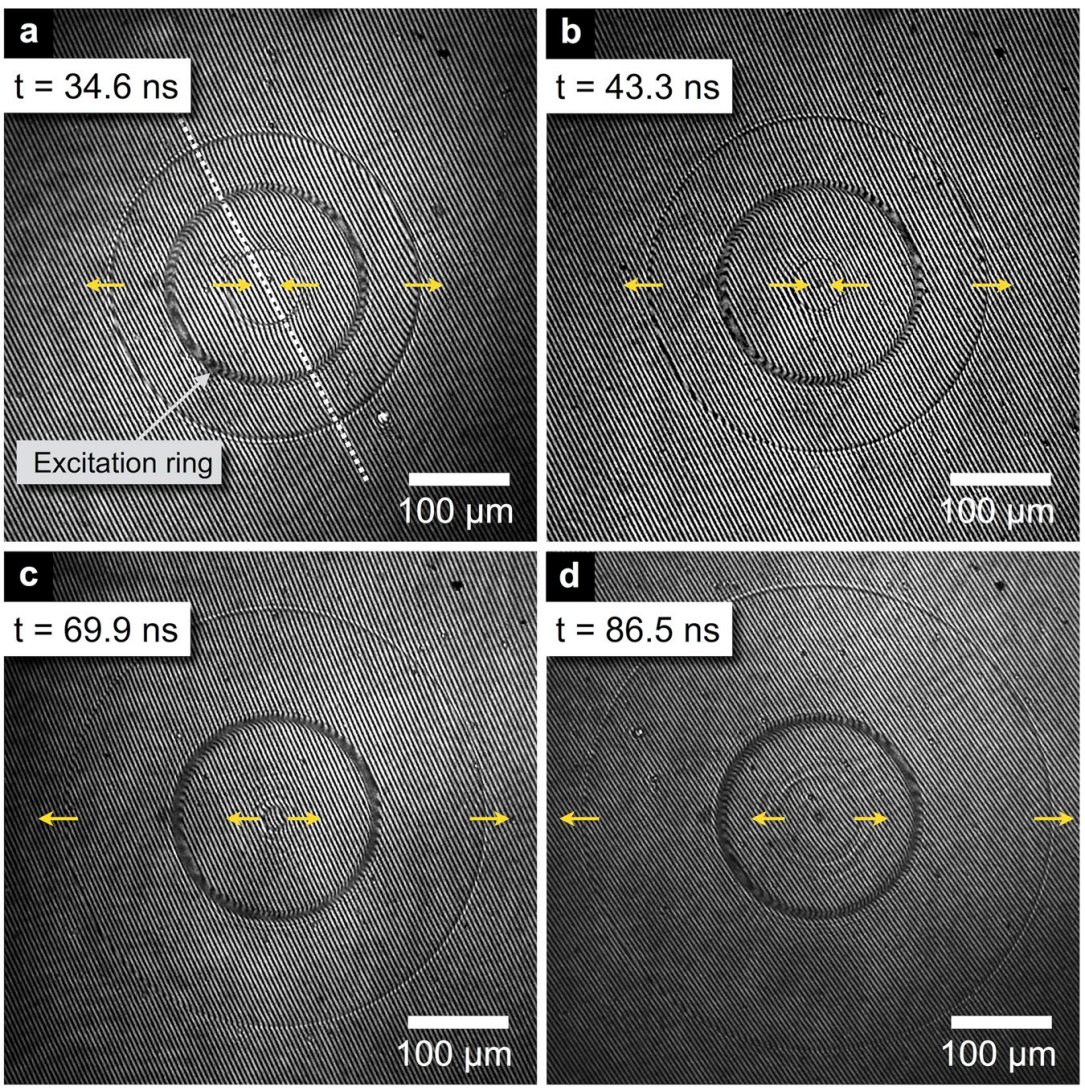
Interferometric images showing shock wave propagation and focusing produced by 0.05 mJ excitation pulse for 4 different delays. Arrows indicate the propagation direction of the shock fronts. (**c**,**d**) The shock that was converging in (**a**,**b**) is now diverging from the center. Bubbles are formed at the laser irradiated area and appear as a black ring on the images. The fringe density is about 200 periods/mm.

A typical set of interferometric images taken with increasing probe pulse delays at an excitation pulse energy of 0.05 mJ is shown in Fig. 2. The shock fronts manifested themselves through a phase jump, or a rapid fringe bend. For a 0.05 mJ excitation energy, the shock wave propagating inward focused for about 60 ns (Fig. 2a,b) when it reached the center and then re-emerged as a diverging wave (Fig. 2c,d).

**Figure 3:**
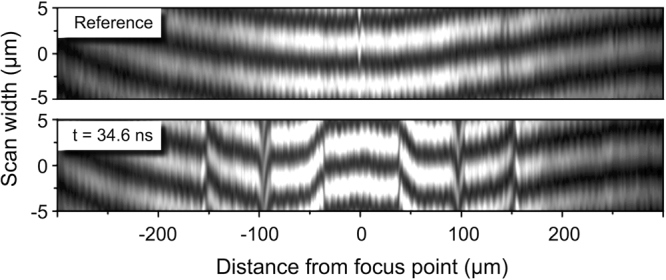
Interference fringes extracted along a diameter from a reference image taken few seconds before shock generation (top) and the image from Fig. 2a with a 34.6 ns delay (bottom).

To quantitatively analyze the images, a narrow strip (10 µm wide) was selected along a diameter of the image, as shown in Fig. 2a, and the spatial phase of the interference fringe pattern was extracted within the strip by fitting the intensity distribution to a sinusoidal functional form (Fig. 3). A reference phase was extracted from a reference image taken few seconds before laser excitation and shock generation. The reference phase was then subtracted from the phase measured on the shock image. A constant phase difference between the shock image and the reference image caused by vibrations of optical elements, e.g., mirrors or beamsplitters, was eliminated by setting the phase shift in the undisturbed area outside the shock to zero. From the phase shift, the refractive index variation Δ*n* was calculated and then translated into the density *ρ* using the empirically-determined formula for water^26^, valid under shock conditions for densities ranging from to 1.00 to 1.21 g/cm^3^:

1 $${\rm{\Delta }}n=0.322\times \rho \,[g/c{m}^{3}]$$

It is important to mention that upon fitting the phase across a shock front, a multiple of 2π uncertainty arises and therefore the phase difference from the reference can only be determined within a multiple of 2π. To overcome this problem, it is necessary to take a series of images, at a given delay, with small excitation energy increments which leads to phase increments smaller than 2π in order to obtain unambiguous values of the phase for a given excitation energy.

**Figure 4:**
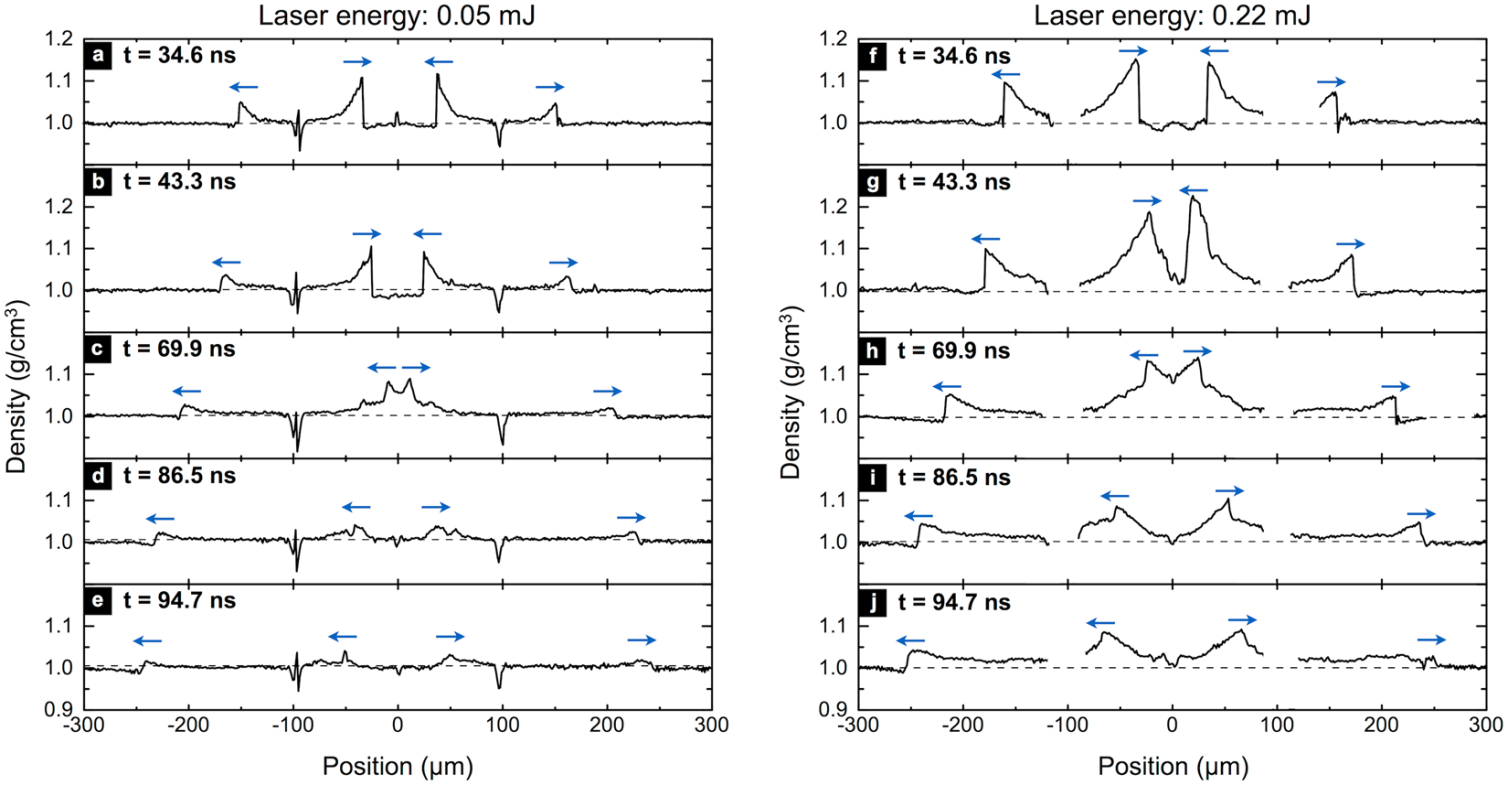
Water density profiles extracted along a ring diameter for five delays between the excitation pulse and the probe pulse: 34.6 ns, 43.3 ns, 69.9 ns, 86.5 ns, and 94.7 ns for laser excitation energies of (**a–e**) 0.05 mJ and (**f–j**) 0.22 mJ. (**a–d**) Correspond to the images shown in Fig. 2. Rapid density jumps indicate shock fronts, with the arrows showing the propagation direction. The horizontal dashed line marks the undisturbed density of water at room temperature. The density drop at x = ±95 µm is caused by bubble formation at the excitation ring location (**a–e**). A density dip at the center in (**d,e**,**i**,**j**) indicates the formation of a cavitation bubble.

The calculated density profiles for two laser excitation energies of 0.05 mJ (corresponding to the four images shown in Fig. 2) and 0.22 mJ and for an additional delay of 94.7 ns are presented in Fig. 4. As stated in the previous section, the inward-propagating wave converged toward the center (Fig. 4a,b,f,g) and was then observed as a diverging wave after 69.9 ns (Fig. 4c–e,h–j).

The greater density, measured for an excitation energy of 0.05 mJ, at the converging shock front compared to the diverging shock at 34.6 ns, 0.11 ± 0.03 g/cm^3^ compared to 0.05 ± 0.02 g/cm^3^ corresponding to pressures of 3.3 ± 1.2 kbar and 1.2 ± 0.6 kbar respectively, attests to the shock pressure enhancement though 2D focusing. The diverging wave decreased in amplitude because of the combined effects of attenuation and cylindrical divergence. Shock pressures associated with the measured density jumps at the shock front were calculated using the Tait type equation of state of water^27^. The uncertainty in the liquid layer thickness (~20%) represented the main source of uncertainty for the density calculation which directly affected the uncertainty of the estimated pressures. It would be straightforward to reduce this uncertainty by measuring the layer thickness at the exact location of (and immediately prior to) the shock measurement.

## Discussion

Because the impedance mismatch between the liquid and the substrates is not infinite, a stress waves is expected to be generated in the glass substrates upon laser absorption in the liquid layer. However, under the present weak-shock situation, the stress wave in the glass did not induce a phase shift that was significant enough to be detected above the noise level of the phase measurement. Nevertheless, at higher laser energies (40 times higher), the wave in the glass can be observed, such as in ref. 13, and is easily distinguishable from the shock in the liquid because of the propagation speed difference.

Absolute shock pressures are commonly extracted from shock speeds using the Hugoniot data^28,29^. We estimated the speeds of the shock fronts for both the diverging and focusing shock waves at 34.6 ns by extracting the shock propagation distances from the image. For an excitation energy of 0.05 mJ, the measured speeds corresponded to 1-D shock pressure^30^ of 2.9 ± 1.5 kbar and 1.7 ± 0.5 kbar for the focusing and diverging waves, respectively, as calculated from the following Hugoniot data for water^28^:

2 $$P={\rho }_{0}\,{u}_{S}\,\frac{{u}_{S}-{c}_{0}}{1.99}$$

where *u*_*S*_ is the shock speed, *c*_*0*_ is 1.45 km/s (acoustic velocity in water) and *ρ*_*0*_ is 0.998 g/cm^3^ (density of the undisturbed water at room temperature). The pressures calculated from the density and speed measurements agreed within the respective uncertainties for both laser excitation energies used (see Table 1). We also note good agreement between the laser fluence dependence on the shock pressure observed here and the dependence measured in ref. 13.

**Table 1:** Comparison of shock front pressure values obtained from the present interferometric method and from speed measurements, after a propagation time of 34.6 ns.

	Converging shock	Diverging shock
**Laser energy: 0.05 mJ**		
Using interferometric method	3.3 kbar ± 1.2 kbar	1.2 kbar ± 0.6 kbar
Using speed measurements	2.9 kbar ± 1.5 kbar	1.7 kbar ± 0.5 kbar
**Laser energy: 0.22 mJ**		
Using interferometric method	5.2 kbar ± 1.9 kbar	2.9 kbar ± 1.0 kbar
Using speed measurements	4.4 kbar ± 1.4 kbar	3.0 kbar ± 1.3 kbar

It is also worth noting that there is a tendency for pressures values obtained for the converging shocks using the interferometric method to be higher than those obtained by speed calculations and inversely for the diverging shock (Table 1). Indeed, because the shock speed was averaged over the propagation distance, it underestimated the instantaneous speed of the converging – therefore accelerating – shock, which resulted in an underestimation of the pressure. The inverse is true for the diverging shock. A better evaluation of shock pressures using shock speeds would hence require instantaneous measurement of the shock front speed, which is particularly difficult when the speed varies rapidly as in the focusing case and when there is a significant interval between measurement times. Streak camera measurements can overcome this difficulty by resolving shock trajectories on a single shot basis with picosecond resolution^13^. In the interferometric method, absolute values of the density, assuming a priori knowledge of the *n*( *ρ*) relation, across the entire sample is directly measured at a single pump-probe delay with no need for additional measurements, given prior determination of the local sample thickness.

**Figure 5:**
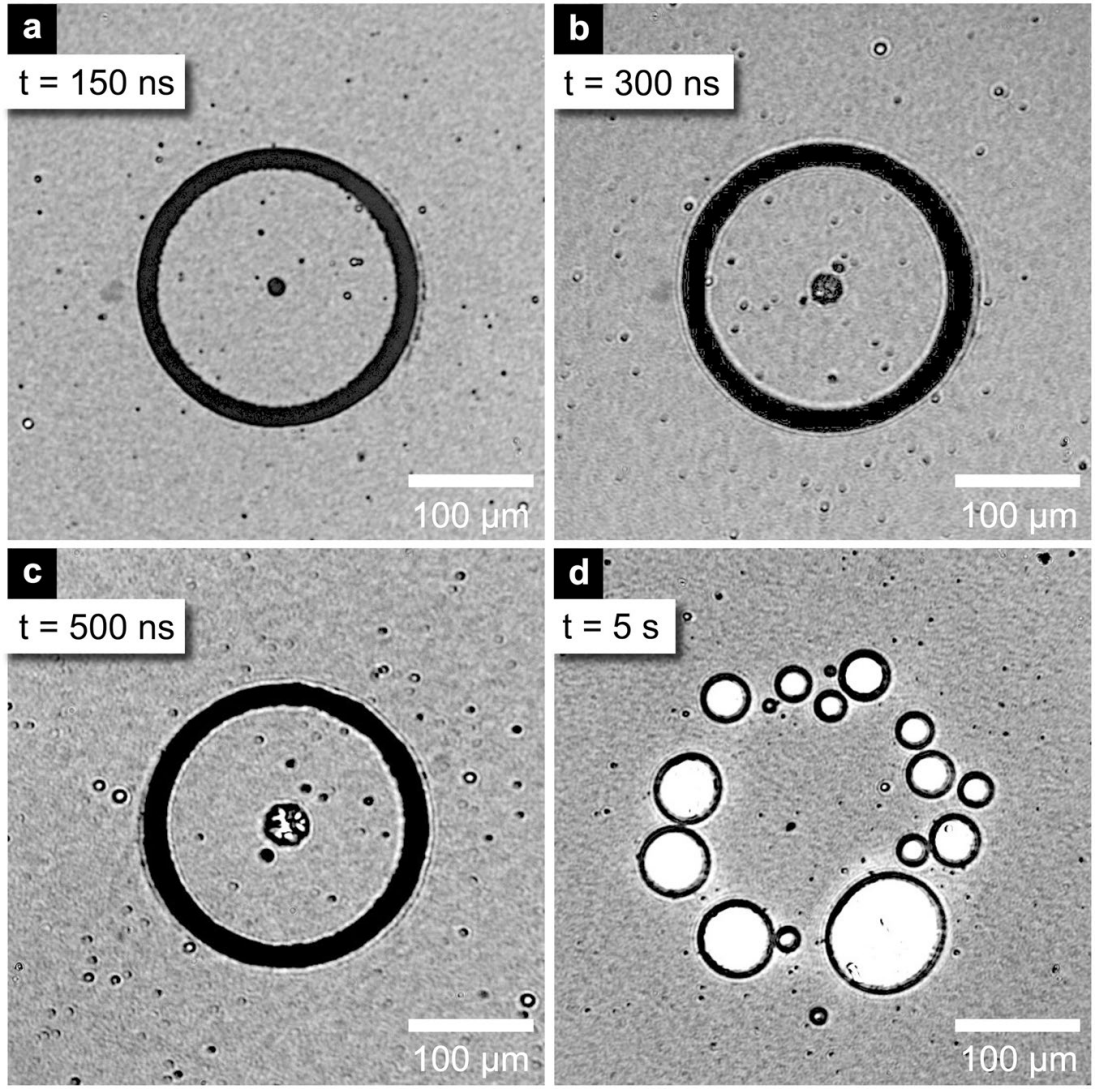
Non-interferometric images recorded (**a**) at 150 ns, (**b**) 300 ns, (**c**) 500 ns and (**d**) at 5 s delay, at the excitation pulse energy of 0.05 mJ.

The local low density at *x* = ±95 µm, shown in Fig. 4a–e, is due to the bubble formation at the laser excitation ring. After the passage of the shock wave through the center, negative pressure developed at *x* = 0 µm (Fig. 4e,i) as a tensile strain followed the shock focus and caused cavitation^31^. The cavitation is a consequence of the Gouy phase shift, a well–known effect that has also been observed through imaging of converging terahertz waves^32^ and surface acoustic waves^33,34^. The bubble formed at the center a few nanoseconds after the shock focus remained for hundreds of nanoseconds before collapsing. The bubble appears in the non-interferometric images shown in Fig. 5a–c, which were recorded for longer delays by using a multimode optical fiber to transmit and delay the probe pulse. While the bubble at the center expanded for hundreds of nanoseconds before collapsing, the laser-induced bubbles generated along the excitation ring persisted for minutes, as shown in Fig. 5d taken few seconds after the shock generation.

In summary, we have used a short laser pulse focused into a ring to generate a cylindrical shock wave in a 10 μm-thick layer of water. Interferometric imaging was used to observe the shock wave as it converged, passed through the center and then diverged, leaving behind a cavitation bubble. We have developed a methodology for the quantitative analysis of interferometric images that allows us to measure material density profiles with a spatial resolution of a few microns. The approach can provide quantitative density changes in new materials under shock conditions if coupled with precise measurement of the layer thickness and knowledge of the density-dependent refractive index. In addition to simple liquids, our experimental geometry can be used to study the effect of shock waves and shock-induced cavitation on biological cells. This methodology is also applicable to measurements of transparent bulk solid samples under shock loading. Interferometric density measurement is a first step toward developing a range of spectroscopic techniques for local characterization of materials under shock loading with high spatial and temporal resolution. Optical probes in a wide range of spectral regions from THz to UV may be used for vibrational and electronic spectroscopy, and it will be important to know the instantaneous local density with which any spectral changes should be associated.

## Methods

### Sample preparation

The liquid sample was made by diluting black Indian ink (Majuscule®) 10× in water to yield a ~2 wt% carbon concentration. The liquid was enclosed between two 300 µm-thick, 1 inch-diameter, glass substrates (Schott D263®). A photoresist (SU-8 2005, MicroChem®) was coated and developed on one of the substrate to form a ring-patterned spacer. The spacer had an outer diameter of 22 mm, an inner diameter of 19 mm, and a thickness of 10 µm to ensure separation between the substrates.

### Sample characterization

The thickness of the liquid layer was measured using an interferometric technique that relies on the interferences of reflections from the two inner faces of the glass windows enclosing the liquid layer, also called Haidinger fringes^35^. Measurements on a number of samples prepared in the same way yielded an average thickness of 10.6 µm with a standard deviation of 2.8 µm. The absorbance of the liquid layer was measured using a UV-VIS-NIR Spectrophotometer. 99% of the excitation light (800 nm wavelength) was absorbed by the carbon particle suspension.
